# Total Knee Arthroplasty After Genicular Nerve Radiofrequency Ablation: Reduction in Prolonged Opioid Use Without Increased Postsurgical Complications

**DOI:** 10.5435/JAAOSGlobal-D-22-00125

**Published:** 2022-08-12

**Authors:** Seth Stake, Amil R. Agarwal, Stefan Coombs, Jordan S. Cohen, Gregory J. Golladay, Joshua C. Campbell, Savyasachi C. Thakkar

**Affiliations:** From the Department of Orthopaedic Surgery, George Washington School of Medicine and Health Sciences (Dr. Stake, Agarwal, Dr. Campbell); the Department of Orthopaedic Surgery, Howard University Hospital (Dr. Coombs), Washington, DC; the Department of Orthopaedic Surgery, Penn Medicine, Philadelphia, PA (Dr. Cohen); the Department of Orthopaedic Surgery, VCU Health, Richmond, VA (Dr. Golladay); and the Johns Hopkins Department of Orthopaedic Surgery, Adult Reconstruction Division, Columbia, MD (Dr. Thakkar).

## Abstract

Genicular nerve radiofrequency ablation (GNRFA) is an increasingly used nonsurgical treatment modality for patients with advanced knee osteoarthritis. Previous studies have demonstrated this to be an effective and safe method to decrease pain and increase functionality in this patient population. The purpose of this study was to compare 2-year postoperative complication rates and rates of prolonged postoperative opioid usage between patients undergoing total knee arthroplasty (TKA) after previous GNRFA and those undergoing TKA alone.

**Methods:**

Patients who underwent primary TKA after prior GNRFA (GNRFA-TKA) of the ipsilateral knee were identified in a national all-payer claims database from 2010 to 2019. Univariate and multivariable analyses were conducted comparing those with prior GNRFA and those without. Outcomes of interest included prolonged postoperative opioid usage, 2-year revision rates, and 90-day medical complications. Statistical analysis was conducted using R software provided by the PearlDiver Database.

**Results:**

In total, 675 patients in the GNRFA-TKA cohort were compared with a control cohort of 255,351 patients. Genicular nerve radiofrequency ablation-total knee arthroplasty patientshad lower odds of prolonged opioid use postoperatively (OR: 0.478; 95%: 0.409 to 0.559; *P* < 0.001). No notable difference was observed in the 2-year surgical outcomes between cohorts. Patients in the GNRFA-TKA cohort had lower odds of requiring a blood transfusion and having postoperative anemia, all arrhythmias, and urinary infections compared with primary TKA control patients.

**Conclusion:**

Preoperative GNRFA leads to a lower rate of prolonged postoperative opioid use in patients undergoing TKA, without an increased risk of complications. Future prospective studies are needed to validate the findings of this database study.

Total knee arthroplasty (TKA) is the procedure of choice for end-stage knee osteoarthritis (OA) that has failed nonsurgical treatment,^1,2^ and most patients are satisfied with their outcomes.^3^ Nonsurgical interventions, including weight loss, medication, therapeutic exercise, and possibly corticosteroid injection, are recommended for knee OA^4,5^ to ameliorate symptoms and to facilitate recovery after surgery.

Other modalities such as electrical stimulation and ultrasonography can be administered for knee OA; however, the evidence is not strongly in favor of these options. More recently, genicular nerve radiofrequency ablation (GNRFA) has gained popularity in the management of both preoperative and postoperative knee pain. Genicular nerve radiofrequency ablation is a two-step procedure that involves a diagnostic extra-articular lidocaine block, followed by targeted thermal ablation to the superior lateral, superior medial, and inferior medial genicular sensory nerve branches of the knee.^6^ In the most recent American Academy of Orthopaedic Surgeons Clinical Practice Guidelines for nonsurgical management of knee OA, treatments classified as denervation therapy received a limited recommendation as a nonsurgical treatment modality for patients with symptomatic OA.^5^

Recent studies have sought to define the effectiveness of GNRFA and to compare it with other nonarthroplasty treatments for knee osteoarthritis. A high-quality, double-blind, sham-controlled study showed that cryoneurolysis targeting the infrapatellar branch of the saphenous nerve resulted in reduced pain and improved osteoarthritis symptoms.^7^ Other studies have demonstrated RFA to be an effective modality to decrease pain and increase functionality in patients with OA.^8–11^ In addition, two RCTs comparing intra-articular (IA) corticosteroids with GNRFA have shown favorable pain and function scores for ablation within the first three months and persisting to at least 6 months.^12,13^ The evidence for RFA is well summarized in a recent systematic review, which concluded that RFA has superior results to NSAIDs and IA corticosteroids with no serious adverse events reported.^2^

Although GNRFA is an increasingly used effective modality to temporize OA symptoms, it has not been shown to slow the degenerative process. Thus, patients with severe OA who have previously undergone GNRFA may progress to TKA. There is currently a paucity of literature on adverse events and outcomes associated with TKA after previous GNRFA (GNRFA-TKA). Although limited to studies with small patient cohorts of less than 100 patients, two prospective trials have not found benefit for RFA in controlling pain after TKA.^14,17^ However, adverse postoperative complications were not compared across cohorts in these studies. Other nonsurgical modalities, such as IA corticosteroid injections, have been correlated with a time-dependent increased risk for postoperative complications after TKA.^16^ It is important to identify procedure-related TKA risks so that patients undergoing GNRFA-TKA can be properly counseled. The purpose of this study was to compare (1) two-year postoperative complication and revision rates and (2) the rate of prolonged opioid use between patients undergoing GNRFA-TKA and those undergoing primary TKA without previous nerve ablation using a national all-payer claims database. Time interval between GNRFA and TKA was also noted because intra-articular steroid injection timing has been related to TKA complications.^16^ We hypothesized that GNRFA-TKA patients would have no difference in complication and revision rates as well as a reduced incidence of prolonged opioid use compared with primary TKA control patients.

## Methods

A retrospective cohort analysis was conducted using data from the PearlDiver Patients Records Database (www.pearldiverinc.com). The Mariner data set was used in this study and includes all-payer claims data from 2010 to 2019. The data set is unique in that it longitudinally follows patients based on distinct patient identifiers, minimizing loss to follow-up in the system. Patients who underwent primary TKA were identified using International Classification of Diseases 10 procedure codes (ICD-10). Patients who underwent primary TKA for diagnoses other than osteoarthritis were excluded from this study. Records of patients meeting the inclusion criteria were queried to identify those who underwent GNRFA before TKA. Patients who underwent GNRFA were identified using the CPT code 64640. The Mariner data set of PearlDiver does not have CPT modifiers to control for laterality. To confirm that GNRFA and TKA were conducted on the ipsilateral side, ICD-10 diagnosis codes were used. An ICD-10 diagnosis code of OA with controlled laterality was associated with the CPT code for GNRFA. Only those with GNRFA before TKA and with matching laterality were included in our GNRFA-TKA cohort.

### Exclusion and Inclusion Criteria

Patients were excluded if they were younger than 18 years at the time of TKA, had a staged or simultaneous bilateral TKA within our follow-up period, or had a postoperative nerve ablation procedure. Staged/bilateral TKAs were excluded to control for laterality of the revision outcomes. Patients were included if they underwent TKA for OA and had a follow-up of 2 years within the Mariner data set. Patients without two years of follow-up, as determined using the patient manifest, were excluded and were assumed to be either lost to follow-up or to have had their procedure after 2017 and thus did not have 2 years of follow-up by the end of our study period. Owing to the limitations of ICD-10 code availability and the requirement for 2-year follow-up data, this study ultimately included patients who underwent TKA from 2015 to 2017.

### Demographics and Outcome Variables

Demographic characteristics collected included age, sex, and the Elixhauser comorbidities for each cohort.^17^ Preoperative opioid use was defined as having had an opioid medication prescription filled at least once within one year before TKA. The primary outcomes for this study were 2-year surgical outcomes (all-cause revision, revision for periprosthetic joint infection [PJI], revision for loosening, and manipulation under anesthesia [MUA]) and postoperative prolonged opioid use. Prolonged opioid use was defined as continued opioid use, prescribed in the postoperative period, within the 3- to 6-month postoperative window after TKA. By three months postoperatively, most patients without preoperative opioid use no longer require opioids for the management of their postoperative TKA pain.^18^ Opioid use was tracked only through 6 months postoperatively to prevent any overlap from any future procedures that required postoperative opioid prescriptions or other unrelated sources of pain. Secondary outcomes included 90-day readmissions and various 90-day medical complications including surgical site infections, postoperative anemia, bleeding complications/transfusions, other infectious complications, deep vein thrombosis, pulmonary embolism, and death.

### Statistical Analysis

Univariate analysis using the R software (R Foundation for Statistical Computing) provided by PearlDiver was used to analyze any differences in patient demographics, comorbidities, complications, and outcomes. This was conducted using chi square tests for categorical variables and Student *t*-tests for continuous variables where appropriate. To mitigate confounding variables and covariates, all demographic variables and comorbidities with *P*-values less than 0.2 on univariate analysis were included as independent variables for each multivariable analysis. All outcomes and complications with *P*-values less than 0.2 were included as dependent variables for separate multivariable analyses. Using the PearlDiver software, logistic regression was conducted for multivariable analysis of the indicated variables. A *P*-value of less than 0.05 was used as the level of significance. For confidentiality purposes, PearlDiver does not permit the reporting of count data less than 11. Any count data with a patient number less than 11 was reported as <11, but the appropriate percentage was reported.

## Results

A total of 255,910 patients who underwent TKA for osteoarthritis were included in this study. Of these patients, 675 underwent GNRFA before TKA (GNRFA-TKA) and 255,351 did not undergo GNRFA before TKA. For those who underwent a prior GNRFA, the average time between GNRFA and TKA was 96.39 days (SD: 91.23).

### Demographics and Comorbidities

Those with prior GNRFA were older (*P* < 0.001); more likely to be smokers (*P* < 0.001); and more likely to have congestive heart failure (*P* = 0.038), arrhythmias (*P*< 0.001), peripheral vascular disease (*P* < 0.001), neurological disorders (*P* = 0.002), chronic pulmonary disease (*P* = 0.005), diabetes mellitus (*P* = 0.017), hypothyroidism (*P* = 0.039), liver disease (*P* = 0.044), collagen vascular disorders (*P* < 0.001), fluid and electrolyte disorders (*P* < 0.001), blood loss anemia (*P* = 0.011), iron deficiency anemia (*P* = 0.004), drug abuse (*P* < 0.001), psychoses (*P* < 0.001), depression (*P* < 0.001), obesity (*P* < 0.001), and preoperative opioid use (*P* < 0.001) when compared with those without prior GNRFA (Table 1).

**Table 1:** Demographics and Comorbidities of Nerve Ablation Before TKA and Control-TKA

Category	Total	GNRFA-TKA		Control-TKA		
	Number	Number	Percent	Number	Percent	*P*
Total	255,910	675	—	255,351	—	**<0.001**
Age						
<50	-	<11	1.19	23,817	20.02	—
50-59	51,303	174	25.78	51,129	34.01	—
60-69	87,103	260	38.52	86,843	36.64	—
70-80	93,795	233	34.52	93,562	20.02	—
Sex						
Male	94,359	236	34.96	94,123	36.86	0.593
Female	161,666	439	65.04	161,227	63.14	0.593
CHF	37,705	119	17.63	37,586	14.72	**0.038**
Arrhythmias	102,908	314	46.52	102,594	40.18	**<0.001**
Valvular disease	58,306	173	25.63	58,133	22.77	**0.084**
Pulmonary circulatory disorders	21,140	57	8.44	21,083	8.26	0.915
PVD	58,591	203	30.07	58,388	22.87	**<0.001**
HTN	182,699	492	72.89	182,207	71.36	0.402
Paralysis	5,469	16	2.37	5,453	2.14	0.773
Other neurological disorders	19.156	72	10.67	19,084	7.47	**0.002**
CPD	92,974	281	41.63	92,693	36.30	**0.005**
Diabetes mellitus	96,941	286	42.37	96,655	37.85	**0.017**
Hypothyroidism	79,535	235	34.81	79,300	31.06	**0.039**
CKD	47,298	135	20.00	47,163	18.47	0.33
Liver disease	41,419	136	20.15	41,283	16.17	**0.006**
PUD	7,469	29	4.30	7,440	2.91	**0.044**
CVD	47,639	246	36.44	47,393	18.56	**<0.001**
Coagulopathy	19,406	60	8.89	19,346	7.58	0.225
Fluid and electrolyte disorders	87,382	273	40.44	87,109	34.11	**<0.001**
Blood loss anemia	13,532	51	7.56	13,481	5.28	**0.011**
Deficiency anemia	45,361	149	22.07	45,212	17.71	**0.004**
Alcohol abuse	—	<11	1.04	2778	1.09	1.000
Drug abuse	19,708	127	18.81	19,581	7.67	**<0.001**
Psychoses	7,632	38	5.63	7,594	2.97	**<0.001**
Depression	100,202	330	48.89	99,872	39.11	**<0.001**
Smoking	43,889	150	22.22	43,739	17.13	**<0.001**
Obesity	122,019	398	58.96	121,621	47.63	**<0.001**
Preoperative opioid use	108,007	325	48.15	107,682	42.17	**0.002**

CHF = congestive heart failure, CKD = chronic kidney disease, CPD = chronic pulmonary disease, CVD = collagen vascular diseases, GNRFA = genicular nerve radiofrequency ablation, HTN = hypertension, PUD = peptic ulcer disease, PVD = peripheral vascular disease, TKA = total knee arthroplasty

Significance at *P* < 0.05 is presented in bold.

### Univariate Postoperative Complications and Revisions

The GNRFA-TKA cohort had significantly lower prolonged opioid usage (50.81% versus 56.29%; *P* = 0.005), postoperative anemia (3.70% versus 13.70%; *P* < 0.001), atrial fibrillation (5.78% versus 8.29%; *P* = 0.022), blood transfusion requirement (1.04% versus 2.24%; *P* = 0.047), and urinary tract infection (3.85% versus 8.47%; *P* < 0.001) when compared with those without prior GNRFA (Table 2).

**Table 2:** Univariate Postoperative Outcomes and Complications of Nerve Ablation Before TKA and Control-TKA

Category	Total	GNRFA-TKA		Control-TKA		
	Number	Number	Percent	Number	Percent	*P*
Total	255,910	675	—	255,351	—	**-**
Prolonged opioid use	144,080	343	50.81	143,737	56.29	**0.005**
2-yr outcomes						
All-cause revision	6279	16	2.37	6263	2.45	0.989
PJI-indicated revision	—	<11	1.19	1990	0.78	0.328
Loosening-indicated revision	—	<11	0.59	1653	0.65	1.000
MUA	8,477	19	2.81	8,458	3.31	0.539
90-day complications						
SSI	4,716	11	1.63	4,705	1.84	0.789
Renal failure	10,355	18	2.67	10,337	4.05	0.085
Anemia	35,010	25	3.70	34,985	13.70	**<0.001**
Arrhythmia with afib	21,218	39	5.78	21,179	8.29	**0.022**
Arrhythmia without afib	10,291	13	1.93	10,278	4.03	**0.007**
Bleeding complication	—	<11	0.89	1,634	0.64	0.57
Blood transfusion	—	<11	1.04	5,730	2.24	**0.047**
Pneumonia	5,934	14	2.07	5,920	2.32	0.769
Stroke	—	<11	1.04	2,867	1.12	0.978
Death	34	0	0.00	34	0.01	1
DVT	—	<11	0.44	3,559	1.39	0.053
Heart failure	14,250	35	5.19	14,215	5.57	0.728
Pulmonary embolism	—	<11	1.19	3,864	1.51	0.59
Respiratory complication	—	<11	0.15	1,965	0.77	0.104
Sepsis	—	11	1.63	4,470	1.75	0.927
UTI	21,651	26	3.85	21,625	8.47	**<0.001**

Afib = atrial fibrillation, DVT = deep vein thrombosis, GNRFA = genicular nerve radiofrequency ablation, MUA = manipulation under anesthesia, PJI = periprosthetic joint infection, SSI = surgical site infection, TKA = total knee arthroplasty, UTI = urinary tract infection

Significance at *P* < 0.05 is presented in bold.

### Multivariable Postoperative Complications and Revisions

The GNRFA-TKA cohort had significantly lower odds of prolonged opioid usage (OR: 0.478; 95%: 0.409 to 0.559; *P* < 0.001), postoperative anemia (OR: 0.170; 95%: 0.111 to 0.249; *P* < 0.001), atrial fibrillation (OR: 0.517; 95%: 0.366 to 0.731; *P* < 0.001), arrhythmias without atrial fibrillation (OR: 0.274; 95%: 0.157 to 0.478; *P* = 0.005), blood transfusion requirement (OR: 0.338; 95%: 0.144 to 0.662; *P* < 0.001), and urinary tract infection incidence (OR: 0.250; 95%: 0.166 to 0.366; *P* < 0.001) when compared with those without prior GNRFA (Table 3).

**Table 3:** Multivariable Postoperative Outcomes and Complications of Nerve Ablation Before TKA and Control-TKA

Category	Total	GNRFA-TKA	Control-TKA	
	Odds Ratio	95% CI		*P*
Anemia	0.170	0.111-0.249		**<0.001**
Atrial fibrillation	0.517	0.366-0.731		**<0.001**
Arrhythmia without atrial fibrillation	0.274	0.157-0.478		**<0.001**
Blood transfusion	0.338	0.144-0.662		**0.005**
UTI	0.250	0.166-0.366		**<0.001**
Prolonged opioid use	0.478	0.409-0.559		**<0.001**

GNRFA = genicular nerve radiofrequency, TKA = total knee arthroplasty, UTI = urinary tract infection.

Ablation significance at *P* < 0.05 is presented in bold.

## Discussion

This study was a retrospective cohort analysis using a national all-payers claims database comparing patients who were treated with genicular nerve radiofrequency ablation before TKA (GNRFA-TKA) with primary TKA control patients. Prolonged postoperative opioid use was found to be markedly lower in GNRFA-TKA patients, although markedly more of these patients had filled an opioid prescription within one year preoperatively compared with control patients. No differences were observed in the primary two-year outcomes (all-cause revision, revision for infection, revision for loosening, and MUA) between patients who underwent prior GNRFA and those who did not. Regarding the secondary outcome of 90-day postoperative complications, GNRFA-TKA patients had lower rates of blood transfusions, anemia, arrhythmias, and urinary tract infections compared with control patients. No other notable differences were observed in other surgical outcomes between cohorts. The association between GNFRA and these statistically significant variables is uncertain. However, the baseline characteristics of the GNFRA cohort suggest that these patients might have been predicted to be at higher risk of complications.

In the era of the current opioid epidemic, there has been a call for alternative nonopioid treatment options for knee OA.^2,19^ GNRFA has demonstrated notable efficacy and safety in the nonsurgical management of knee OA.^12,13,20,24^ Cooled radiofrequency thermal ablation is an FDA-approved treatment of chronic moderate-to-severe knee pain caused by OA. High-quality RCTs have shown favorable outcomes with GNRFA knees when compared with IA HA, IA corticosteroids, standard oral nonopioid analgesics, and sham procedures.^13,20,22,24^ At this time, the American Academy of Orthopaedic Surgeons Clinical Practice Guidelines endorse limited evidence in favor of denervation procedures for knee OA.^5^ However, the effect of these procedures on complications and pain after TKA remains poorly understood.

Genicular nerve radiofrequency ablation is generally regarded as a safe procedure, with complications occurring rarely and including hypoesthesia, numbness, septic arthritis, and pes anserine injury.^25–28^ No study to date has investigated the difference in postoperative complications in TKA patients who had previously undergone GNRFA versus patients who had not undergone GNRFA. We hypothesized that GNRFA-TKA patients would have similar rates of 2-year complications and a lower incidence of prolonged opioid use compared with primary TKA control patients.

This study found no increased rate of MUA or revisions, including revision for loosening or infection, at 2 years postoperatively in patients who underwent prior GNRFA. On average, GNFRA preceded TKA by 96.39 days, but the standard deviation was 91.23 days, suggesting that some patients may have had the procedure as part of a preemptive analgesic modality in the perioperative period. This is an important finding of our study because a previous study has shown a time-dependent increased risk of postoperative infection in patients who undergo TKA within 3 months of prior steroid injection.^29^ GNRFA does not seem to pose the same increased infection risk.

Our study found decreased odds of anemia, postoperative blood transfusions, arrhythmias, and urinary tract infections in patients who underwent GNFRA before TKA. These differences in postoperative complications are not clear, although several factors may be contributory. Given that the GNRFA patients had some increased comorbid factors and GNFRA requires specialized expertise, equipment, and resources, it is possible that the GNFRA cohort may have had their surgery at a tertiary care or specialty facility. These centers may have been more likely to routinely use evidence-based blood management programs including increased use of preoperative optimization protocols, appropriate transfusion thresholds, neuraxial anesthesia, and TXA administration in this patient cohort, strategies which have been shown to decrease blood loss and risk of transfusion.^30^ In addition, patients with defined pathways at tertiary centers may be better optimized for weight loss, smoking cessation, and perioperative interventions. Although not included in this multivariable analysis, high-volume centers have been reported to have improved complications after total knee arthroplasty procedures.^31,32^ Laucis et al^31^ identified that the odds ratio for complications in low-volume hospitals compared with very high-volume hospitals was 1.327 (95% CI 1.26 to 1.40; *P* < 0.0001).

Predicting and preventing prolonged opioid use in total knee arthroplasty patients has been a focus of recent studies, with the most important risk factor of prolonged use being preoperative use.^33,34^ Although GNRFA has consistently been shown to be effective, resulting in improvement in WOMAC scores for pain stiffness and function, previous published results have been variable and less favorable regarding its effectiveness on opioid consumption.^11,35,36^ In a randomized clinical trial comparing GNRFA with hyaluronic acid injection for chronic knee pain, Chen et al demonstrated a decreased total daily dose of nonopioid medications after 6 months in the cooled GNRFA group but were unable to measure any trends in opioid consumption.^2^ Notably, less than 10 patients in each arm of this study were on opioid medications, which limited the study's power to detect differences in this outcome.^2^ In a prospective, randomized, sham-controlled trial evaluating the efficacy of GNRFA 2 to 6 weeks before elective TKA on postoperative pain outcomes, they noted no measurable effect on postoperative opioid use, analgesia use, or function in the 48 hours after surgery.^15^ In addition, in that study, there was a low incidence of preoperative opioid use and nearly complete opioid weaning by 6 months in all patients, regardless of the treatment group.^15^ As noted by the authors, the trends in opioid use in that trial may have reflected prescribing patterns of physicians rather than patient preferences.^15^ By comparison, opioid usage both before and after TKA was much higher in this database study, which increases the generalizability of our findings. Patients who underwent GNRFA before TKA had a markedly lower rate of prolonged postoperative opioid use after multivariable analysis (OR 0.478; 95% CI 0.409 to 0.559). This suggests that there may be long-lasting analgesic effects of GNRFA, persisting into the postoperative period. Notably, preoperative opioid use was shown to be markedly more frequent in patients who underwent GNRFA before TKA.

There are several limitations to our study. Retrospective analysis of a claims database relies on coding accuracy, which may be prone to error. Genicular nerve radiofrequency ablation is a novel nonsurgical treatment option requiring specialized expertise and equipment, which may create selection bias. However, the study group had higher rates of comorbid conditions, which partly mitigates this potential bias. The cohort receiving GNFRA may more likely have had care provided at tertiary-level centers or as part of a comprehensive arthroplasty program. These centers are more likely to medically optimize patients in anticipation for a total knee arthroplasty potentially leading to a decreased PJI rate in the GNRFA patient cohort. Another limitation in this study is the lack of uniformity in the GNRFA procedures. We acknowledge that there are substantial variations in the mode of ablation (cold vs conventional), techniques (variations in nerves targeted), and selection criteria for this treatment modality. Although this study specified the nerve ablation procedure to be indicated for knee OA, it relied on the use of CPT 64640, which nonspecifically encompasses all the earlier mentioned techniques. Another limitation is that the study design could not consider any differences in the postoperative analgesic protocol after TKA in both groups, including whether multimodal therapy including anti-inflammatory medications, acetaminophen, and gabapentinoids were used. Finally, the study design did not account for institutional differences in opioid prescribing practices after surgery.

## Conclusion

GNFRA before TKA is associated with a lower risk of prolonged opioid use, without an increased risk of complications, including PJI. Future prospective randomized controlled cohort studies are necessary to confirm these findings.
